# Examining the Relationship Between Pediatric Behavioral Health and Parent Productivity Through a Parent-Reported Survey in the Time of COVID-19: Exploratory Study

**DOI:** 10.2196/37285

**Published:** 2022-08-18

**Authors:** David Grodberg, Jesse Bridgewater, Theoren Loo, Dena Bravata

**Affiliations:** 1 Child Study Center Yale University School of Medicine New Haven, CT United States; 2 Brightline Palo Alto, CA United States; 3 Center for Primary Care and Outcomes Research Stanford University Palo Alto, CA United States

**Keywords:** adolescent, child, family health, mental health, behavioral health, stress, protective factors, productivity, COVID-19

## Abstract

**Background:**

Pediatric behavioral health needs skyrocketed during the COVID-19 pandemic. Parents and caregivers lacked access to well-established tools to identify risk and protective factors while also experiencing decreased access to treatment options to meet their families’ behavioral health needs.

**Objective:**

The aim of this study is to investigate the associations of known pediatric behavioral health risk factors and parents’ reports of workplace productivity.

**Methods:**

A clinical research team at Brightline—a virtual, pediatric behavioral health solution—drew on standardized instruments to create a survey designed to understand pediatric behavioral health conditions, child stress, and family resilience and connection during the COVID-19 pandemic. Multivariable linear regression was used to characterize the relationship between these variables and parents’ reports of workplace productivity.

**Results:**

Participants (N=361) completed the survey between October 2020 and November 2021. In the multivariable model, higher pediatric stress and time spent managing children’s behavioral health needs were associated with greater productivity loss among working parents, whereas higher family connection was associated with lower productivity loss. COVID-19 diagnoses among parents and dependents, financial impact of COVID-19 on households, and family resilience were not associated with parents’ workplace productivity.

**Conclusions:**

This survey captured child stress, family connection, and productivity as reported by parents and caregivers during the COVID-19 pandemic. Exploratory studies are the first step in understanding the relationship between these variables. The results from this study can empower parents by providing insights to help manage their child’s behavioral health concerns and identify pediatric behavioral health services to aid working parents who are caregivers.

## Introduction

In March 2020, the COVID-19 pandemic was declared in the United States, which set off a flurry of lockdowns, school closures, and layoffs. The pandemic amplified the already increasing prevalence of mental health conditions for both adults and children, and exacerbated barriers to receiving behavioral health services [1-4]. While the risks for mental health disorders associated with the pandemic have broadly risen, they have disproportionately affected children and adolescents who already experience even greater barriers to care than adults [5].

As a result, rates of depression and anxiety among children have doubled since the pandemic began. Currently, 1 in 4 children experience increased depression symptoms, and 1 in 5 experience increased anxiety symptoms [6]. Adolescent girls, in particular, have visited emergency departments during the pandemic for eating disorders and tic behaviors at alarming rates [7,8]. Not surprisingly, the decline in children’s mental health and well-being during the pandemic is now identified as a major cause of parents’ increased stress, job turnover, decreased productivity, and lost income [9].

The prolonged and far-reaching impact of pandemics such as COVID-19 on well-being requires not only increased surveillance of pediatric mental health disorders but also identification of child- and household-level risk and protective factors that may affect pediatric health outcomes [10,11]. For instance, studies have identified risk factors for disaster-related mental health problems among children and adolescents [12-14]. Such factors include both nonmodifiable and modifiable factors, including gender (female), age (younger), ethnic minority, preexisting disabilities, predisaster emotional status, previous trauma history, postdisaster levels of psychological distress, and family resilience [12]. Knowing risk and protective factors can help to predict who may be vulnerable to future disaster-related mental health conditions.

However, because childhood is a time of change and development, it can be challenging for parents to gauge how they and their children are doing with respect to these known risk factors [15]. As such, parents and caregivers often rely on input from schools, day cares, and other caregivers to help them notice potentially problematic shifts and changes in their child’s behavior. During calamities such as the COVID-19 pandemic, parents may be cut off from these community resources and support. The lack of access to these support systems and mental health services during the recent pandemic-related closures led to decreased mental health screenings, diminished ability to identify learning disabilities, and less child-protective and mental health referrals [16].

Delays in addressing modifiable risk factors for pediatric behavioral health problems over the past 2 years of COVID-19–related shutdowns have resulted in higher rates of emergency department visits attributed to mental health concerns among children and adolescents [17]. The emergency medical services system, though, is not the optimal point of access for effective treatment due to (1) a shortage of on-site mental health professionals to accurately diagnose or treat conditions, (2) long waits to receive care, and (3) patients failing to continue treatment postdischarge [18]. What follows is a cycle of crisis care, which is ultimately costly, ineffective, and more time intensive than early prevention and intervention. This cycle of crisis care for youth in distress arguably leads to increased parental stress as their children remain undiagnosed or ineffectively treated.

For employed parents, balancing the demands of raising children with behavioral health needs against work commitments can lead to higher levels of stress and burnout, making it difficult to be a present parent and productive employee—which were amplified when our country overwhelmingly pivoted to work from home [19,20]. During lockdowns and school closures, parents and caregivers lacked access to easy-to-use tools to help identify their children’s risk for mental and behavioral health conditions as well as resources to help them manage their children’s behavioral health outside of the emergency department. Our clinical research team at Brightline—a virtual behavioral health solution designed for children (aged 18 months up to 18 years) and their families—recognized this gap in support and saw an immediate need to help parents and caregivers who were struggling to understand and meet the mental and behavioral needs of their children.

As a response, we developed a web-based 30-item survey to assess 4 key areas linked to increased risk of behavioral health conditions among children and adolescents: preexisting behavioral health conditions, psychological stress, family resilience, and family connection. As a mental and behavioral digital health company that provides services to children and their families, we were interested in understanding the broad impact that child behavioral health conditions have on other aspects of a parent’s life. Therefore, we added questions to ascertain how parents perceived their child’s behavioral health impacted their productivity at work. Given the context of the global pandemic, we also included standardized questions about direct household impacts of COVID-19 as they relate to finances and COVID-19 diagnoses of household members.

This exploratory study of survey responses investigated the associations of pediatric behavioral health risk factors and parents’ workplace productivity. While the survey was developed during the COVID-19 pandemic, we were not researching the direct impacts of COVID-19 on psychological stress, children’s behavioral health conditions, or time spent managing behavioral health. Instead, our aim in creating the survey was to provide parents with feedback on their child’s mental and behavioral health based on their survey responses, and to guide them to relevant resources.

## Methods

### Recruitment

Brightline is a company that offers a technology-enabled behavioral health solution designed for children (aged 18 months up to 18 years) and their families [21]. Brightline delivers self-guided content, coaching services, and virtual care through multidisciplinary care teams, family-focused support, and evidence-based care delivery geared toward helping children and their families across developmental stages. Commonly reported behavioral health needs by Brightline users include anxiety, depression, and disruptive behavior disorders.

The web-based survey was available on a landing page on the Brightline company website between October 2020 and November 2021 and provided an overview of the survey to participants (Multimedia Appendix 1). A link to the survey was shared on Brightline’s social media platforms, such as LinkedIn and Twitter, throughout the survey window. Other than being shared on social media and through the other avenues, participants were not actively recruited to participate in the survey. Moreover, Brightline membership was not required to access and complete the survey.

### Participants

Anyone who visited the Brightline website during the study period could complete the survey. Participants were not compensated for completing the survey. There were no other eligibility criteria or exclusion criteria for participation. While this allowed our survey to be accessible to any interested participant, it also meant that a nonparent could complete the survey.

### Procedures

Participants voluntarily completed the anonymous, web-based survey. The survey took approximately 10 minutes to complete. Once participants submitted the survey, they received a summary of each of the 4 survey components (Multimedia Appendix 2). The participant could then choose to follow a link to resources and a guide to help facilitate a conversation with a pediatric behavioral health care provider (Multimedia Appendices 3 and 4).

### Measures

The survey was designed from 3 standardized clinical instruments, focusing on psychological stress, family resilience, family connection, and preexisting behavioral health conditions. The survey was augmented by a set of questions about parental productivity and time spent managing children’s behavioral health, as well as questions related to the direct impacts of the COVID-19 pandemic and participant demographics (Multimedia Appendix 5). These instruments were selected because they aligned with previous research that identified both nonmodifiable and modifiable risk factors for children’s postdisaster mental health outcomes and well-being, such as mass disasters (eg, 9/11 attacks), natural disasters (eg, hurricanes), and previous epidemics [11-14]. Further, these instruments were selected because they do not evaluate for any clinical domains that would require Brightline to intervene in the case of high acuity needs.

#### Preexisting Behavioral Health Conditions

Preexisting behavioral health conditions were assessed with children with special health care needs (CSHCN) screener [19]. The CSHCN screener is a set of 5 yes or no question sequences used to identify children with special health care needs (Multimedia Appendix 5). The purpose of including the CSHCN screener was not to evaluate whether COVID-19 was a catalyst for behavioral health conditions in the participants’ children but rather to assess for already existing behavioral health conditions.

#### Psychological Stress

Pediatric stress was measured during the pandemic, specifically at the time of the survey, using the PROMIS (Patient-Reported Outcome Measurement Information System) Pediatric Parent Proxy Psychological Stress Experiences Measure (Multimedia Appendix 5) [22]. PROMIS has been used in research to study children’s stress levels associated with cancer treatment, sickle cell disease, as well as other chronic illnesses [23-27]. Participants rated the frequency of their child’s stress experience on a 5-point Likert scale. Scores were grouped into 4 categories based on general interpretation guidelines: scores less than 50 indicate pediatric stress was within normal limits; scores between 50 and 55 indicate mild pediatric stress; scores between 55 and 65 indicate moderate pediatric stress; and scores greater than 65 indicate severe pediatric stress [22].

#### Family Resilience and Connection Index

The family resilience and connection index (FRCI) was used to measure family resilience and connection, a 6-item index that comprises 4 family resilience items and 2 additional items that measure parent-child connection and parent coping (Multimedia Appendix 5) [28]. The FRCI has been used in research to evaluate the connection between family resilience and parenting stress with children’s mental health and attention deficit hyperactivity disorder as well as their ability to flourish and engage at school [29-31]. The 4-item family resilience index (FRI) asked parents about their approach to problem-solving and hopefulness. Additionally, the 2-item family connection index (FCI) asked parents about their connectedness with children and perception of how well they are managing the day-to-day demands of child raising. One point was assigned for each time a participant answered “all of the time” to 1 of the 4 FRI items. Moreover, one point was assigned for each time a parent responded “very well” to 1 of the 2 FCI items. Scores were grouped into three categories based on general interpretation guidelines: scores between 0 and 1 indicate low family resilience or connection; scores between 2 and 3 indicate moderate family resilience or connection; and scores between 4 and 6 indicate strong family resilience or connection.

#### Productivity

Two additional questions written by the research team were added about time spent by parents managing their children’s behavioral health concerns, and about its impact on productivity (Multimedia Appendix 5).

#### COVID-19 Impact

To determine the potentially adverse effects COVID-19 may have had on participants, a set of 3 yes or no questions from the Coronavirus Aid, Relief, and Economic Security Act was included in the survey (Multimedia Appendix 5) [32]. These questions asked whether the respondent experienced a COVID-19 adverse event, which includes being diagnosed with COVID-19, a spouse or dependent being diagnosed, or the presence of an adverse financial consequence from COVID-19. The questions about the pandemic were included to ascertain COVID-19 exposure experiences as a facet of participant demographics.

#### Background and Demographics

The following demographics information was collected: marital status of the parent answering the survey, household income, race or ethnicity, highest educational level achieved, gender, and state of residence (Multimedia Appendix 5).

### Analyses

This retrospective, cross-sectional study analyzed survey responses collected from participants (N=361) between October 2020 and November 2021. All statistical analyses were performed in R version 4.1.2 (R Foundation for Statistical Computing). Prior to analysis, all predictor variables underwent min-max normalization. Multivariable linear regression was used to characterize the associations among productivity interruptions, family stress, family connection, family resilience, and COVID-19–related exposure experiences. This linear regression was adjusted for household income and parental education level. Goodness of fit was performed for linear regressions.

### Ethics Approval

This study protocol was approved by the Western Institutional Review Board and the Copernicus Group Institutional Review Board.

## Results

### Participants

Participant demographics are presented in Table 1. The participants primarily had a White racial background, were married women who were highly educated, and reported high incomes.

**Table 1:** Participant demographics (N=361).

Variable		Value, n (%)
**Gender**		
	Female	257 (71.1)
	Male	62 (17.2)
	Nonbinary	10 (2.8)
	Prefer not to say	32 (8.9)
**Marital status**		
	Divorced	22 (6.1)
	Married	278 (77.0)
	Prefer not to say	9 (2.5)
	Separated	21 (5.8)
	Single, never married	19 (5.3)
	Widowed	12 (3.3)
**Education level**		
	Completed some high school	7 (1.9)
	High school graduate	19 (5.3)
	Associate degree	18 (5.0)
	Completed some college	32 (8.9)
	Bachelor’s degree	87 (24.1)
	Completed some postgraduate	21 (5.8)
	Master’s degree	123 (34.1)
	PhD, law, or medical degree	33 (9.8)
	Prefer not to say	21 (5.8)
**Household income (US $)**		
	<25,000	19 (5.3)
	25,000 to 35,000	13 (3.6)
	35,001 to 50,000	20 (5.5)
	50,001 to 75,000	36 (10.0)
	75,001 to 100,000	37 (10.2)
	100,01 to 150,000	50 (13.9)
	>150,000	147 (40.7)
	Prefer not to say	39 (10.8)
**Race or ethnicity**		
	American Indian or Alaska Native	7 (1.9)
	Asian	45 (12.5)
	Black or African American	24 (6.6)
	Hispanic or Latino	31 (8.6)
	Native Hawaiian or Other Pacific Islander	2 (0.5)
	Other or multi-ethnic	15 (4.2)
	Prefer not to say	40 (11.1)
	White	197 (54.6)

### COVID-19 Exposure-Related Experiences

The direct impact of COVID-19 on participants are presented in Table 2. Over 90% (341/361) of the participants reported neither they nor their spouse had been diagnosed with COVID-19. The responses to the question “I have experienced adverse financial consequences due to COVID-19” are also summarized in Table 2. A majority of participants (233/361, 65%) said they had not experienced financial consequences as a result of COVID-19.

**Table 2:** COVID-19 exposure-related experiences (N=361).

Question	“Yes,” n (%)	“No,” n (%)
I was diagnosed with COVID-19.	20 (6)	341 (94)
My spouse or my dependent was diagnosed with COVID-19.	26 (7)	335 (93)
I have experienced adverse financial consequences because (1) I or a member of my household was quarantined, laid off, or had work hours reduced due to COVID-19; (2) I or a member of my household was unable to work as a result of a lack of childcare due to COVID-19; (3) a business owned or operated by me or a member of my household closed or reduced hours due to COVID-19; or (4) I or a member of my household had a reduction in pay (or self-employment income) due to COVID-19 or had a job offer rescinded or start date for a job delayed due to COVID-19.	128 (35)	233 (65)

### Survey Results: CSHCN Screener Results

The preexisting behavioral health needs of the participants’ children from the CSHCN screener, which is commonly used to evaluate whether a child already has behavioral health needs prior to evaluation, are presented in Table 3. Approximately 28% (102/361) were described as having an emotional, developmental, or behavioral problem for which he or she needs or gets treatment or counseling, and 21% (76/361) reported their child needing more medical care, mental health, or educational services than is usual for most children.

**Table 3:** CSHCN^a^ screener results.

Question	“Yes,” n (%)	“No,” n (%)
Does your child currently need or use medicine prescribed by a doctor (other than vitamins)?	51 (14)	309 (86)
Does your child need or use more medical care, mental health, or educational services than is usual for most children of the same age?	76 (21)	285 (79)
Is your child limited or prevented in any way in his or her ability to do the things most children of the same age can do?	57 (16)	303 (84)
Does your child need or get special therapy, such as physical, occupational, or speech therapy?	56 (16)	304 (84)
Does your child have any kind of emotional, developmental, or behavioral problem for which he or she needs or gets treatment or counseling?	102 (28)	258 (72)

^a^CSHCN: children with special health care needs.

The responses for the item “Since Covid began, how much time (on average) are you spending managing your child or children’s behavioral health concerns (including their stress, anxiety, disruptive behaviors)?” are summarized in Table 4. Nearly 50% (155/361) of the participants reported spending between 2 and 4 hours per week managing their children’s behavioral health. The responses to the question “How much is your child’s behavioral health and well-being affecting your productivity and ability to work if you are currently employed?” are also summarized in Table 4. The majority of the participants (307/361, 85%) reported some impact on their productivity and ability to work.

The responses for the PROMIS measure are summarized in Table 5. The mean summed PROMIS raw score was 23.05. The scaled score is approximately 64.4, which indicates moderate pediatric stress reported at the time of the survey among the participants in our study.

The responses for the FRCI measures are summarized in Table 6. The mean summed FRI score was 1.03, indicating low family resilience among participants in our study. The mean summed FCI score was 2.3, indicating moderate family connection among the participants in our study.

**Table 4:** The impact of child’s behavioral health on parents’ time and productivity.

Variable		Value, n (%)
**Time managing child behavioral health concerns results (hours/week)**		
	0-1	78 (22)
	2-4	155 (43)
	5-8	77 (21)
	>8	51 (14)
**Child’s behavioral health affecting your productivity results**		
	Large impact	34 (9)
	Definite impact	144 (40)
	Slight impact	130 (36)
	No impact	53 (15)

**Table 5:** PROMIS^a^ results.

Variable	Mean (SD)
PROMIS pediatric physical stress raw summed score	23.05 (6.15)
In the past 7 days, my child felt overwhelmed.	3.05 (0.87)
In the past 7 days, my child felt that his or her problems kept piling up.	2.76 (1.05)
In the past 7 days, my child felt that he or she had too much going on.	2.66 (1.06)
In the past 7 days, my child felt unable to manage things in his or her life.	2.68 (1.01)
In the past 7 days, my child felt under pressure.	2.80 (0.99)
In the past 7 days, my child had trouble concentrating.	3.14 (1.05)
In the past 7 days, everything bothered my child.	2.72 (1.03)
In the past 7 days, my child felt stressed.	3.23 (0.83

^a^PROMIS: Patient-Reported Outcome Measurement Information System.

**Table 6:** FRCI^a^ results.

Variable		Mean (SD)
**Family resilience index score**		1.03 (0.52)
	When your family faces problems, how often are you likely to talk together about what to do?	0.56 (0.36)
	When your family faces problems, how often are you likely to know we have strengths to draw on?	0.57 (0.35)
	When your family faces problems, how often are you likely to work together to solve our problems?	0.60 (0.29)
	When your family faces problems, how often are you likely to stay hopeful even in difficult times?	0.56 (0.31)
**Family connection index score**		2.30 (1.00)
	How well can or do you think you are managing the day-to-day demands of raising children?	0.48 (0.28)
	How well can or do you share ideas and talk about things that really matter with your child?	0.55 (0.32)

^a^FCRI: family resilience and connection index.

### Factors Associated With Productivity Loss

In the multivariable model, there was a strong association between productivity loss and the time spent managing children’s behavioral health needs (β=.365; *P*<.001) as well as Stress Index scores (β=.213: *P*<.001) (Table 7). There was a weaker association between productivity loss and FCI scores (β=–.125; *P*=.053) (Table 7). COVID-19 diagnoses among parents and dependents, financial impact of COVID-19 on households, and family resilience were not associated with parents’ workplace productivity (Table 7).

**Table 7:** Multivariable linear regression of survey items associated with productivity loss (F^9^_351_=14.97; *P*<.001; adjusted R^2^=0.2588).

Independent variables	β	SE β	*P* value
Time spent managing child behavioral health concerns	.365	.043	<.001
PROMIS^a^ pediatric physical stress raw summed score	.213	.075	<.001
Family connection score	–.125	.064	.053
Family resilience score	–.019	.067	.78
COVID-19 diagnosis (self)	–.099	.068	.14
COVID-19 diagnosis (partner or dependent)	.036	.060	.54
Adverse COVID-19 financial impact	.025	.029	.34
Caregiver education	–.015	.056	.79
Household income	.032	.024	.18

^a^PROMIS: Patient-Reported Outcome Measurement Information System.

## Discussion

### Principal Findings

Our findings show that parents spend a considerable amount of time managing their children’s behavioral health needs. Although we did not find that having a COVID-19 diagnosis among a household member or having a significant financial impact on the household from COVID-19 impacted productivity, this may have been due to the fact that our participants had low rates of COVID-19 infection and were relatively high-income earners. An important extension of this work would be to assess the association of COVID-19 diagnoses and financial consequences of the pandemic on workplace productivity among lower-income earners and those with significant COVID-19–associated illness.

In addition, we found that high pediatric stress and more time spent managing children’s behavioral health problems were associated with increased productivity losses for parents regardless of COVID-19–related experiences. While COVID-19 variables were used as predictors in our multivariable regression model, we discovered that COVID-19 exposure-related experiences did not have a significant effect on our outcome. Despite the lack of representation of families affected by COVID-19, we felt it was important to incorporate this variable in our model.

Our findings also showed that enhanced parent-child connection is inversely associated with the parents’ productivity loss, even amid adversity [33-35]. This finding is significant because there are known behavioral health interventions that can be mobilized to enhance parent-child connection where parents are trained to support the management of children’s behavioral health problems. Our results further support those of previous studies, which show that pediatric stress and family connection are good predictors of pediatric behavioral health outcomes [36-38].

### Limitations and Future Studies

As with all survey research, there are limits inherent to this research method. As the survey was completed anonymously and was internet-based, we were unable to control for the chance that a nonparent completed the survey. Moreover, the tool produced some basic pediatric behavioral health insights that were tailored to the users’ answers after completing the survey; therefore, the participants may have been encouraged to take the survey more than once in order to receive different answers. This means that the same participant could have taken the survey multiple times without us knowing. However, allowing for anonymity likely increased the response rate and participation.

In this exploratory study, the majority of respondents represented a well-educated, high-income, female population. Further, participants were only recruited using digital channels (eg, search engines, email, and social media), all of which can potentially limit the generalizability to other populations. The participants may have included parents searching on the web for information related to child behavioral health; therefore, their responses related to perceived child stress and family resilience might not reflect the general parent population. Future studies will share the survey through alternate venues to recruit more diverse participants.

In designing the survey, we did not include questions on the number, age, and gender of children, potentially limiting the ability to draw insights on the differences between children and adolescents across behavioral health needs and impact on stress, family resilience, and family connection. However, research has shown that having a higher number of children is positively associated with a higher level of psychological distress in families [39]. Additionally, the survey did not include questions from the Sickness Index Profile, which evaluates the effect of disease on physical and emotional functioning [40]. There is opportunity to further explore the impact of COVID-19 and other illnesses or stressors on children’s behavioral health needs.

Building on our preliminary findings, in future studies, we aim to learn more about the time spent and its impacts on the larger family unit. In this iteration of the survey, we asked parents about their time spent managing their child’s behavioral health concerns. What we received is an estimated snapshot. We are interested in developing questions and methods to ascertain changes in time spent to learn whether this is associated with differing productivity and stress levels. Further, this survey includes responses from parents and caregivers on their individual time spent managing their children’s behavioral health (independent of their partner). Collecting data on time spend on the household level would give us greater insight into the impact a child’s mental and behavioral health has on the family and measures of productivity more broadly. Lastly, when completing the survey, the participants received recommendations and tools to help support their children’s mental and behavioral health needs. In future studies, we aim to collect data on the use of these resources.

### Conclusion

Our preliminary findings confirm that measures of increased psychological stress in children and lower family connection are associated with productivity loss regardless of COVID-19 diagnosis, financial consequences of COVID-19, education level, and income level. These results suggest a continued need for research on family-focused behavioral health benefits that address pediatric stress levels and that offer support to manage children’s behavioral health problems and improve parent-child connections. Surveys that integrate standardized and validated measures of known risk and protective factors for children’s postdisaster well-being and outcomes can be useful mental and behavioral health points of entry for parents. The feedback that participants receive after completing a survey, for example, can make parents or caregivers more aware of these factors and help them to facilitate conversations with health care professionals about their concerns. This is especially important during crises such as COVID-19, when families have limited access to face-to-face community indicators of well-being.

## Appendix

Multimedia Appendix 1 Survey landing page.

Multimedia Appendix 2 Sample survey results summary.

Multimedia Appendix 3 Resources from survey results.

Multimedia Appendix 4 Guide for finding behavioral health care.

Multimedia Appendix 5 Survey items.

## Abbreviations

COBI: COVID-19 behavioral health instrument
CSHCN: children with special health care needs
FCI: family connection index
FRCI: family resilience and connection index
FRI: family resilience index
PROMIS: Patient-Reported Outcome Measurement Information System
