# Supplementary figures and images for: Examining the Relationship Between Pediatric Behavioral Health and Parent Productivity Through a Parent-Reported Survey in the Time of COVID-19: Exploratory Study
Source: JMIR Form Res. 2022 Aug 18;6(8):e37285. doi: 10.2196/37285 (PMC9390832; doi:10.2196/37285)

### Multimedia Appendix 1: Survey Landing Page

**
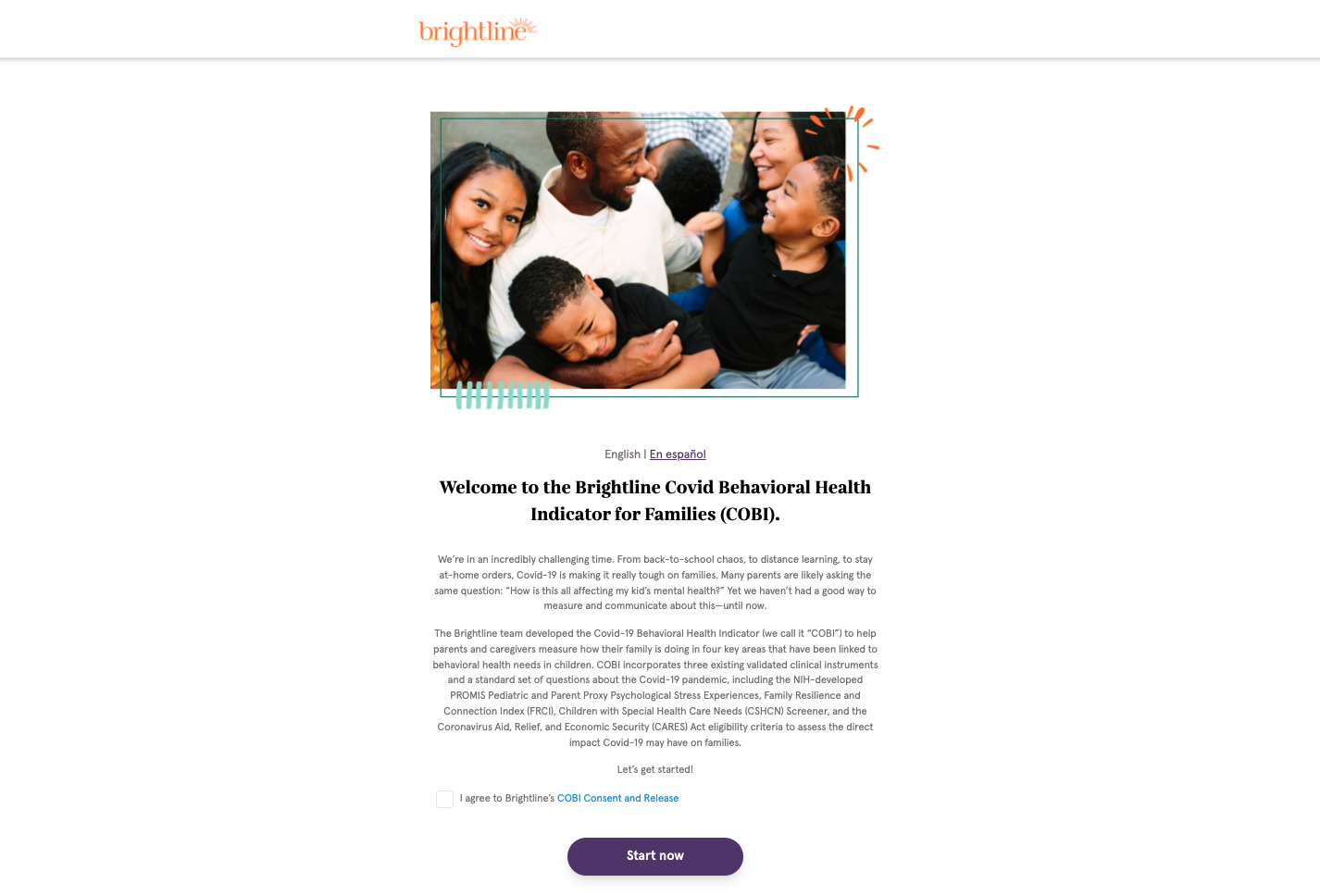
**

Supplement: Multimedia Appendix 1 [file formative_v6i8e37285_app1.docx]

### Multimedia Appendix 2: Sample Survey Results Summary

**
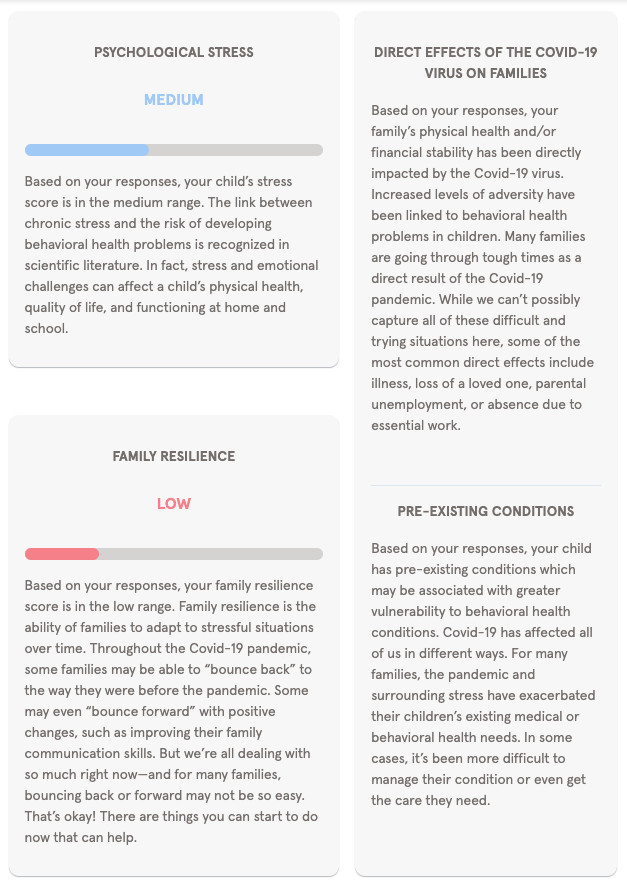
**

Supplement: Multimedia Appendix 2 [file formative_v6i8e37285_app2.docx]

### Multimedia Appendix 3: Resources from Survey Results

**
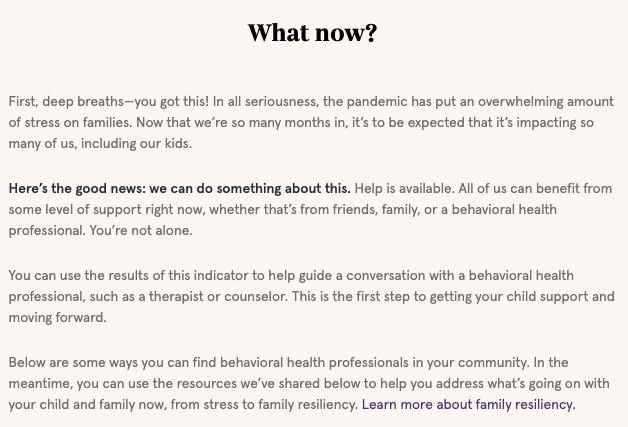
**

Supplement: Multimedia Appendix 3 [file formative_v6i8e37285_app3.docx]

### Multimedia Appendix 4: Guide for Finding Behavioral Health Care

**
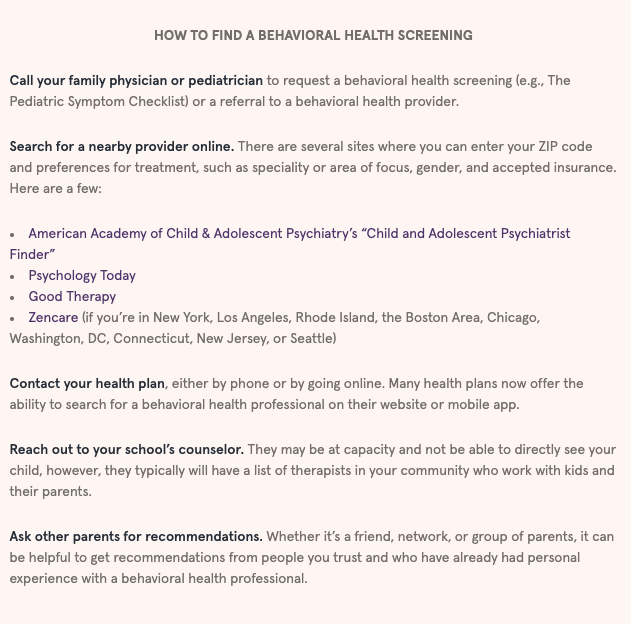
**

Supplement: Multimedia Appendix 4 [file formative_v6i8e37285_app4.docx]
