# Supplementary material for: Examining the Relationship Between Pediatric Behavioral Health and Parent Productivity Through a Parent-Reported Survey in the Time of COVID-19: Exploratory Study
Source: JMIR Form Res. 2022 Aug 18;6(8):e37285. doi: 10.2196/37285 (PMC9390832; doi:10.2196/37285)
Supplement: Multimedia Appendix 5 [file formative_v6i8e37285_app5.docx]

### Multimedia Appendix 5: Survey Items

| Item | Response Option | Source |
| --- | --- | --- |
| I was diagnosed with COVID-19 | Yes or No | Coronavirus Aid, Relief, and Economic Security (CARES) Act [14] |
| My spouse or my dependent was diagnosed with COVID-19. | Yes or No | Coronavirus Aid, Relief, and Economic Security (CARES) Act [14] |
| I have experienced adverse financial consequences because: (i) I or a member of my household was quarantined, furloughed or laid off, or had work hours reduced due to COVID-19;  (ii) I or a member of my household was unable to work due to lack of childcare due to COVID-19;  (iii) a business owned or operated by me or a member of my household closed or reduced hours due to COVID-19; or  (iv) I or a member of my household had a reduction in pay (or self-employment income) due to COVID-19 or had a job offer rescinded or start date for a job delayed due to COVID-19. | Yes or No | Coronavirus Aid, Relief, and Economic Security (CARES) Act [14] |
| When your family faces problems, how often are you likely to talk together about what to do? | None/Some of the time  Most of the time  All of the time | FRCI [11] |
| When your family faces problems, how often are you likely to solve your problems? | None/Some of the time  Most of the time  All of the time | FRCI [11] |
| When your family faces problems, how often are you likely to know you have strengths to draw on? | None/Some of the time  Most of the time  All of the time | FRCI [11] |
| When your family faces problems, how often are you likely to stay hopeful even in difficult times? | None/Some of the time  Most of the time  All of the time | FRCI [11] |
| How well can/do you share ideas and talk about things that really matter with your child? | Not very well or not at all well  Somewhat well  Very well | FRCI [11] |
| How well can/do you think you are managing the day-to-day demands of raising children? | Not very well or not at all well  Somewhat well  Very well | FRCI [11] |
| Does your child currently need or use medicine prescribed by a doctor (other than vitamins)? | Yes or No | CSHCN [11] |
| Is your child limited or prevented in any way in his or her ability to do the things most children of the same age can do? | Yes or No | CSHCN [11] |
| Does your child need or get special therapy, such as physical, occupational or speech therapy? | Yes or No | CSHCN [11] |
| Does your child have any kind of emotional, developmental or behavioral problem for which he or she needs or gets treatment or counseling? | Yes or No | CSHCN [11] |
| Since COVID began, how much time (on average) are you spending managing your child or children's behavioral health concerns (including their stress, anxiety, disruptive behaviors)? | 0-1 hour/week  2-4 hours/week  5-8 hours/week  Greater than 8 hours/week | Researcher generated |
| How much is your child's behavioral health and wellbeing affecting your productivity and ability to work if you are currently employed? | Not at all  Slightly  It's definitely impacting me  It has a large impact on my work productivity | Researcher generated |
| In the past 7 days, my child felt stressed. | Never  Rarely  Sometimes  Often  Always | PROMIS Pediatric Parent Proxy Psychological Stress Experiences Measures [15] |
| In the past 7 days, my child felt that his/her problems kept piling up. | Never  Rarely  Sometimes  Often  Always | PROMIS Pediatric Parent Proxy Psychological Stress Experiences Measures [15] |
| In the past 7 days, my child felt overwhelmed. | Never  Rarely  Sometimes  Often  Always | PROMIS Pediatric Parent Proxy Psychological Stress Experiences Measures [15] |
| In the past 7 days, my child felt unable to manage things in his/her life. | Never  Rarely  Sometimes  Often  Always | PROMIS Pediatric Parent Proxy Psychological Stress Experiences Measures [15] |
| In the past 7 days, everything bothered my child. | Never  Rarely  Sometimes  Often  Always | PROMIS Pediatric Parent Proxy Psychological Stress Experiences Measures [15] |
| In the past 7 days, my child felt under pressure. | Never  Rarely  Sometimes  Often  Always | PROMIS Pediatric Parent Proxy Psychological Stress Experiences Measures [15] |
| In the past 7 days, my child had trouble concentrating. | Never  Rarely  Sometimes  Often  Always | PROMIS Pediatric Parent Proxy Psychological Stress Experiences Measures [15] |
| In the past 7 days, my child felt he/she had too much going on. | Never  Rarely  Sometimes  Often  Always | PROMIS Pediatric Parent Proxy Psychological Stress Experiences Measures [15] |
| Does your child currently need or use medicine prescribed by a doctor (other than vitamins)?   1. Is this because of any medical, behavioral, or other health conditions? 2. Is this a condition that has lasted or is expected to last for at least 12 months? | Yes (go to A) or No (go to next question)   1. Yes (go to B) or No (go to next question) 2. Yes or No | CSHCN Screener [11] |
| Does your child need or use more medical care, mental health or educational services than is usual for most children of the same age?   1. Is this because of ANY medical, behavioral or other health condition? 2. Is this a condition that has lasted or is expected to last for at least 12 months? | Yes (go to A) or No (go to next question)   1. Yes (go to B) or No (go to next question) 2. Yes or No | CSHCN Screener [11] |
| Is your child limited or prevented in any way in his or her ability to do the things most children of the same age can do?   1. Is this because of ANY medical, behavioral or other health condition? 2. Is this a condition that has lasted or is expected to last for at least 12 months? | Yes (go to A) or No (go to next question)   1. Yes (go to B) or No (go to next question) 2. Yes or No | CSHCN Screener [11] |
| Does your child need or get special therapy, such as physical, occupational or speech therapy?   1. Is this because of ANY medical, behavioral or other health condition? 2. Is this a condition that has lasted or is expected to last for at least 12 months? | Yes (go to A) or No (go to next question)   1. Yes (go to B) or No (go to next question) 2. Yes or No | CSHCN Screener [11] |
| Does your child have any kind of emotional, developmental or behavioral problem for which he or she needs or gets treatment or counseling?   1. Has this problem lasted or is it expected to last for at least 12 months? | Yes (go to A) or No (go to next question)   1. Yes or No | CSHCN Screener [11] |
| What is your marital status? | Married  Separated  Widowed  Divorced  Single (never married)  Prefer not say | Demographics |
| What is your annual household income before taxes? | Less than $25,000  $25,000 to $35,000  $35,001 to $50,000  $50,001 to $75,000  $75,001 to $100,000  $100,001 to $150,000  More than $150,000  Prefer not say | Demographics |
| What is your race/ethnicity? | White / Caucasian  Black / African American  Hispanic or Latino  American Indian or Alaska Native  Asian  Native Hawaiian or Other Pacific Islander  Other / Multi-ethnic  Prefer not say | Demographics |
| What is your education level? | Completed some high school  High school graduate  Completed some college  Associate degree  Bachelor's degree  Completed some postgraduate  Master's degree  Ph.D., law or medical degree  Prefer not say | Demographics |
| What is your gender? | Female  Male  Non-binary  Prefer not say | Demographics |
| What state do you live in? | List of all 50 states Prefer Not To Say  Other | Demographics |
